# Mobilising Extremism in Times of Change: Analysing the UK’s Far-Right Online Content During the Pandemic

**DOI:** 10.1007/s10610-023-09547-9

**Published:** 2023-05-30

**Authors:** Jonathan Collins

**Affiliations:** Institute of Political Studies, Faculty of Social Sciences, Charles University, Prague, Czech Republic

**Keywords:** Far-right, Toxic communication, Fringe social media, COVID-19, Mobilisation

## Abstract

The growing dissension towards the political handling of COVID-19, widespread job losses, backlash to extended lockdowns, and hesitancy surrounding the vaccine are propagating toxic far-right discourses in the UK. Moreover, the public is increasingly reliant on different social media platforms, including a growing number of participants on the far-right’s fringe online networks, for all pandemic-related news and interactions. Therefore, with the proliferation of harmful far-right narratives and the public’s reliance on these platforms for socialising, the pandemic environment is a breeding ground for radical ideologically-based mobilisation and social fragmentation. However, there remains a gap in understanding how these far-right online communities, during the pandemic, utilise societal insecurities to attract candidates, maintain viewership, and form a collective on social media platforms. The article aims to better understand online far-right mobilisation by examining, via a mixed-methodology qualitative content analysis and netnography, UK-centric content, narratives, and key political figures on the fringe platform, Gab. Through the dual-qualitative coding and analyses of 925 trending posts, the research outlines the platform’s hate-filled media and the toxic nature of its communications. Moreover, the findings illustrate the far-right’s online discursive dynamics, showcasing the dependence on Michael Hogg’s uncertainty-identity mechanisms in the community’s exploitation of societal insecurity. From these results, I propose a far-right mobilisation model termed *Collective Anxiety*, which illustrates that toxic communication is the foundation for the community’s maintenance and recruitment. These observations set a precedent for hate-filled discourse on the platform and consequently have widespread policy implications that need addressing.

## Introduction

Social media platforms, both mainstream and fringe websites, have become growing mediums for hate speech during COVID-19 (Velásquez et al., 2021). With the dissension towards the pandemic’s political handling, widespread job losses, a backlash to extended lockdowns, and hesitancy surrounding the vaccine, a critical side effect is the propagation of far-right discourse in the UK (Vieten, 2020). Consequently, the right-wing online milieu is radicalising and recruiting followers using the socio-political and economic insecurity that arose during the pandemic (Pantucci & Ong, 2021). For instance, this rise in online extremist activity—an 800% increase in users for a white supremacist network in the USA—is due to the far-right communities’ use of popular and fringe social media platforms (Ackerman & Peterson, 2020). These sites, including Facebook, Twitter, Instagram, YouTube, Gab, and Parler, provide a catalogue of mechanisms for producing and sharing various content forms, subsequently appealing and catering to a vast audience. Notably, the two latter platforms are synonymous with material procured for a far-right-leaning Anglosphere populace (Baines et al., 2021; Jasser et al., 2021; Nouri et al., 2021). Moreover, as recent research suggests, the public is increasingly reliant on social media, including the growing number of participants on fringe sites, for all pandemic-related news and interactions (Cinelli et al., 2020; Neely et al., 2021). Therefore, with the expansion of far-right activities across social media and the public’s reliance on these platforms for socialising, the current pandemic environment is a breeding ground for far-right mobilisation^1^ and toxicity.^2^ Compounding the threat to this expanding network are the real-world societal implications, including the Capitol Hill riot, the rise of QAnon believers and the group’s associated violence, campaigns of targeted hate speech against minorities, and the popularisation of COVID-19 conspiracy theories (Mukhtar, 2021; Munn, 2022; Vieten, 2020).

Scholarly literature on these novel facets of far-right violence, hate-filled online discourse, and societal insecurity have thus far focused on their manifestations across different virtual and offline mediums (Bliuc et al., 2019; Gaudette et al., 2021; Vieten, 2020). However, few have attempted to connect them as one interconnected phenomenon. I aim to solve this gap, providing an in-depth *intragroup* explanation for the toxic online content of this growing movement. Guiding the research is the following question: how does the UK’s far-right online community, during COVID-19, utilise populace anxiety to attract candidates, maintain viewership, and form a collective identity on Gab? By comparing the specific narratives across the UK’s ideological spheres, the findings showcase the commonality of insecurity (vis-à-vis hate-filled narratives) and their subsequent appeal to the growing number of far-right participants. The theoretical foundation of the article is Michael Hogg’s *uncertainty-identity theory*, which aims to be an illustrative model for research on extremist mobilisation (Hogg, 2007, 2009, 2014; Hogg & Adelman, 2013). The collective works outline the relationship between insecurity and extremism, showcasing how the different meso-level group structures within these communities help to reduce anxiety and provide a sense of security. Separated into deductive categories via Hogg’s (2007, 2009) explanatory processes (existential threat, prototypicality, confirmation bias, and entitativity), I demonstrate the popular hate-filled content disseminated on the platform ranging from targeting different out-groups to spreading conspiracies. Notably, these framings have a purpose in solidifying in-group behaviour, ontological worldviews, or prototypical leaders.

Moreover, these mechanisms are not independent but interrelated and help tell a story of co-utilisation leading to the far-right’s increasing online user base. For instance, of the 575 posts connected to confirmation bias narratives, a massive 478 (83%) of these posts also mentioned one of the community’s existential threats. Subsequently, there is a cyclical pattern to this community’s discourse, which relies on both the need and creation of anxiety to recruit followers and solidify their base. This process is perpetually repeated, with new toxic narratives necessary for further mobilisation. I model this recurring pattern as *Collective Anxiety*, a cyclical and dichotomous relationship fuelling societal anxiety while providing answers and a sense of community to manage these uncertainties. Thus, the far-right’s use of toxicity is at the forefront of these findings, a recurring theme observed throughout the article and the basis for discussion.

The study focuses on the relationship between increasing far-right online participation, the community’s toxic social media practices, and the ongoing insecurity-creating dynamics stemming from the pandemic. To provide this connection, the investigation is done through an exploratory framework, employing a mixed-method technique utilising netnography—a reconstituted ethnography for examining social media platforms and their users—and qualitative content analysis (QCA). The social media site, Gab, is selected for its lax moderation policy and ease of accessibility, offering an unrestricted look into different far-right spheres of influence and toxic communication. Moreover, the research uses the UK’s Gab community as a case and boundary for a first-of-its-kind netnographic examination of a fringe platform. Data collection is based on the types of content most attractive to participants during the pandemic. This method involves studying the greater popularity (likes, shares, replies) of specific posts over others (see Hagemann & Abramova, 2023) and categorising them within a deductive codebook. Supplementing the deductive research design are inductive subcategories established by the researcher’s experiential fieldnotes.

### The Far-Right, the UK, Insecurities, and COVID-19

Before the study can approach understanding user mobilisation on Gab, it must first conceptualise and define the term *far-right*. While establishing a consensus definition of the far-right remains a work-in-progress (Pirro, 2022), common facets of the broadly assembled community should best represent the strongest combination for the reader’s understanding. Cas Mudde (2007) provides the most satisfactory and well-regarded fusion of blanket terms for this study, which includes room for the different facets of “family [resemblance]”—extremism, radical, and populist movements—pertinent to showcasing the entire group’s character (Mudde, 2007). As a simplified definition for this article’s investigation, I use Carter’s (2018) amalgamative construction: “an ideology that encompasses authoritarianism, anti-democracy, and exclusionary and/or holistic nationalism”, which leans heavily on the conceptualisation of Mudde and other predominant scholars in the field (for example Eatwell, 2000; Rydgren, 2005). Notably, these scholarly works highlight that the overarching broadness and potential combinations these communities rely upon are typical characteristics of the far-right’s nature. Moreover, researchers conceptualise this ambiguity as a foundation of the group’s persistence. The “cherry-picking” or “mixing and matching” of ideologies from, as Mudde (2002) puts it, “shopping size lists” of possible selections represent the potential variety of the far-right and its various communities, allowing for the group to be adaptive depending on the current environment (Mudde, 2002; Ong, 2020). Thus, this umbrella formulation of the far-right is essential for the study to incorporate the range of ideas, ideologies, content, and discourse proliferating throughout Gab while allowing for potential ideological shifts during the investigation (Pirro, 2022).

Understanding the connection between the far-right’s mobilisation during times of uncertainty requires an outline of the predominant anxieties stemming from the pandemic. What this study calls populace or ontological insecurities (Giddens, 1991)—the omnipresent stress of a changing society, norms, and habits brought about through existential threat—have manifested, both in the UK and globally, in many ways during the pandemic, from immediate health concerns (Grasso et al., 2021; Paul & Vasudevan, 2021; Zoumpourlis et al., 2020) to economic short and long-term effects (Nicola et al., 2020), social dislocation and isolation (Halikiopoulou & Vlandas, 2020; O’Connor et al., 2021), perceived political mishandlings, and frustrations over extended lockdowns (Erhardt et al., 2021; Vieten, 2020; Wondreys & Mudde, 2022). Thus, current insecurity is a direct product of the anxieties caused by the virus (concerning health and safety effects) and is also a by-product of government, institutional, and community responses to the pandemic.

The article starts from the health component, with the immediate concern being the virus’ effects on the individual’s physical condition. As seen by the worldwide tallies of the infected (765 million) and the deceased (6.93 million) at the time of writing, COVID-19 is the most significant viral disease experienced by modern society (WHO Coronavirus, 2023). Notably, the UK has been one of the most affected countries, with over 24.6 million cases and 225 thousand deaths (ibid.). Researchers outline the variety of induced health-related symptoms from psychological (O’Connor et al., 2021; Zoumpourlis et al., 2020) to physical loss and impairment (Grasso et al., 2021) and mortality salience (Paul & Vasudevan, 2021) stemming from the continued viral spread. Under the steady increase of casualties worldwide, societies’ perspectives on the core of life and its nature have become increasingly dismantled, exposing human life as fragile and subject to change. These introspective realisations, in which an individual is aware of an inescapable death, “give rise to significant terror and anxiety” (Paul & Vasudevan, 2021). Not only has this led to increased diagnoses of depression and stress amongst the general population (see Salari et al., 2020), but it has also created an inverse reaction in a significant portion of “non-believers”. These individuals are part of a larger “post-truth” or “anti-science” movement rejecting scientific experts, their findings, and mainstream media reporting on COVID-19’s physical and mental effects. Within this group’s beliefs, health concerns are manufactured as a component of the “Plandemic”—a worldwide elitist scheme falsifying science for personal gain (Prasad, 2022). These attitudes are part of a conspiracy repertoire of far-right content creation targeting the different facets of an insecure society.

Moreover, the pandemic’s socioeconomic impacts penetrate society, affecting individuals regardless of their social or economic status. Some scholars anticipate that, due to the unpredictable nature of COVID-19, the redefinition of work culture, availability, financial cutbacks, and an ultra-competitive job market, the resulting “economic shock” will be the worst seen since WW2 (Grasso et al., 2021). The most pressing result for this study is the uneven distribution of suffering and the deepening of social inequalities for those in a low-strata socioeconomic position (Grasso et al., 2021; Halikiopoulou & Vlandas, 2020). While the pandemic has impacted everyone, those already socially or economically troubled have disproportionately suffered in social mobility, the job market, psychological state (depression, anxiety, and existential fear), and feelings of isolation (Grasso et al., 2021). These effects create socioeconomic vulnerabilities ripe for manipulation, a symptom the far-right exploits to its advantage. For example, a common harmful trope is the concept of victimhood, of being left behind or the “losers of globalisation” (Engler & Weisstanner, 2020).

Another repercussion of the pandemic are the ongoing public rallies and fervent discourse expressing pro and anti-government sentiments. Although political activism has seen some positive demonstrations in favour of government policy (see Wood & Skeggs, 2020), most protests have been negative. Markedly, policies concerning COVID-19—lockdown procedures, mandated masks, travel restrictions, and vaccine administration—continue to be controversial, leading to anger and fear amongst many constituents in the UK and beyond (Erhardt et al., 2021). Compounding these emotions is the perceived lack of good governance within the “new normal” paradigm (Vieten, 2020). Herein, society is losing trust in constantly changing policies they believe are ineffective. Not only do these changes sow inconsistencies and disrupt societal norms, but they continue to breed general uncertainties about future life prospects.

Furthermore, the supposed inability of political parties to provide effective solutions to reduce anxiety, together with the increased fear and anger stemming from this inability, has resulted in some diverging to alternative pathways of political participation (Erhardt et al., 2021). The most observable options are the mainstreaming of radical right politicians, whose platforms are built on many of the abovementioned insecurities. Unsurprisingly, these parties’ platforms range in content depending on the popular grievances exhibited by their constituents while also doubling down on previous hate-filled discourse (ibid.). These messages include accentuating the virus as being of Chinese origin and blaming immigration, criticising mainstream political parties for their ineptness in stopping the pandemic’s spread while also rebutting the “anti-democratic” lockdowns, manipulating events to implicate “enemy” ideologies for spreading the virus, and reinforcing the nature of the post-truth era by undermining mainstream sources (Wondreys & Mudde, 2022). As society’s frustrations grow at the government’s handling of the pandemic, far-right parties and other fringe political movements look more appealing for having the “right” answers. For the far-right, these answers are simple, directed against a scapegoat portion of the population—Muslims, feminist movements, LGBTQ, Jews, leftists—who do not conform to their ultra-conservative values.

How does contemporary research connect the far-right and toxic communications with the above uncertainties, and where does this study’s focus on collectives come into the mix? Several articles highlight the growing hate-filled content across social media during COVID-19 (Caiani et al., 2021; Croucher et al., 2020; Douglas, 2021; Lantz & Wenger, 2023; Vergani et al., 2022). For example, Croucher et al. (2020) emphasise intergroup perceptions of threat, wherein community anxieties redirect into negative out-group stereotyping. Their analysis examines online prejudice against Chinese Americans as the pandemic’s conspiratorial creators and spreaders. Subsequently, Sinophobic hate speech is a common theme (see also Lantz & Wenger, 2023; Sakki & Castrén, 2022; Vergani et al., 2022) for COVID-19 virtual toxicity. Another study details how far-right telegram channels oscillate between narratives, bouncing from Asians as the transmitters of a dangerous health threat to Jewish conspiracies of world domination—a connection to other socio-political and economic anxieties (Vergani et al., 2022). The targets for online toxicity also change depending on the region. For instance, America’s far-right conspiracies concentrate on China’s use of biological warfare, the left’s derailment of Trump’s re-election campaign, and anti-Semitic tropes about utilising lockdowns to control the populace (Douglas, 2021). In the UK, the same inspiration for hate-filled online narratives incorporates anti-Asian discourse (see Sakki & Castrén, 2022) but also includes virtual attacks against the Black Lives Matter movement, political elites, and international health organisations (Caiani et al., 2021). Thus, far-right pandemic narratives are multifaceted and context-dependent. Nevertheless, connecting these findings are the posited intergroup dynamics framed by far-right communities.

Within these pandemic-inspired discursive mechanisms incorporates the recent work on far-right collectives, with authors predominantly focusing on in-group versus out-group framings (Caiani et al., 2021; Douglas, 2021; McNeil-Willson, 2022; Ong, 2020; Sakki & Castrén, 2022). The argument proposes that far-right collectivity depends on inferiorising and dehumanising out-groups to reinforce in-group superiority and survival (Douglas, 2021; Sakki & Castrén, 2022). Herein, far-right virtual spaces mutually develop scapegoats to redirect their frustrations on societal uncertainties. Mcneil-Willson (2022) presents this phenomenon as “out-group community building”, where the movement constructs different antagonists working together to eliminate the in-group. The concept of ideological confluence from Ong (2020) repeats similar findings, where depicting a common enemy helps reaffirm a shared belief system and offers a simple solution—survival is them or us. The many outlined articles examining online Sinophobic hate speech are appropriate for this framing (Gaudette et al., 2021). By dehumanising Chinese, Asians, and other minorities, the far-right collective can rationalise their uncertainties in a simple black-and-white dichotomy, with “us” as the superior and these “others” as the inferior (Sakki & Castrén, 2022). Therefore, out-grouping is an essential aspect of far-right collectivity. However, the concept does not provide a comprehensive framework for understanding collective mobilisation.

The literature highlights two essential elements: (1) the starting point of insecurity on the different societal levels and (2) the end product of far-right out-grouping—and its consequent creation of online hate speech. What it currently fails to answer is the in-between. What narrational elements and intragroup dynamics are happening betwixt these two points leading to far-right mobilisation? I argue that current far-right collectivity is just as much about understanding internal elements as external framings. Thus, we need to improve our understanding of the relationship between the far-right’s utilisation of pandemic-induced societal insecurities and the radicalisation of their ideologies online via in-group mechanisms. In tackling this gap, the article aims to answer how the UK’s far-right community, during COVID-19, utilises populace insecurities to attract candidates, maintain viewership, and form a collective identity on Gab. By providing an in-depth look at the platform’s British-centric content milieu, the study provides a comprehensive investigative tool and narrative connecting the current success of far-right mobilisation with the dynamics of populace insecurity vis-à-vis Michael Hogg’s uncertainty-identity theory framework. Moreover, forming these connections provides the basis for understanding the effectiveness of toxic discourse for the community and its implications for the rest of society.

## Michael Hogg’s Uncertainty-Identity Theory

For the field of extremist research, the theoretical models of social movement (Gunning, 2009) and collective action (Oberschall, 2004) are predominantly employed and reworked to explain the mobilisation of contemporary far-right groups (see Bliuc et al., 2019; Castelli Gattinara & Pirro, 2019; Gaudette et al., 2021; Meadowcroft & Morrow, 2017). Both theories offer a similar formula to understand the appropriate conditions for mobilisation, ranging from discontent and grievances to a shared belief system and the ability to organise (Gunning, 2009; Oberschall, 2004). However, they lack a comprehensive explanation for in-group mechanisms of collectivity. Without this inclusion, the theories struggle to illustrate how insecurities relate to far-right community dynamics. Instead, articles applying social movement or collective action theory often outline particular modules of mobilisation, for example intergroup competition (Bliuc et al., 2019), othering (Gaudette et al., 2021), and positive cost–benefit membership advantages (Meadowcroft & Morrow, 2017). While these studies are crucial for the field, the intragroup nuances for understanding online communities are minimal. How can we comprehend mobilisation without considering the collective itself? I fill this theoretical gap by focusing on the mechanisms promoting group identity, highlighting the deep-seated connection between far-right collectivity and uncertainty.

Michael Hogg’s uncertainty-identity theory (2007) provides a comprehensive argument and explanatory outline to help decipher the intragroup dynamics of far-right online mobilisation during COVID-19. Herein, Hogg unravels the complex paradigm between who we are and how we fit in society, focusing on uncertainty and self-actualisation (Hogg, 2007, 2009, 2020; Hogg et al., 2013). The main argument posits that, through group identity and reinforcement, individuals can remove their insecurity concerning negative or complex world phenomena, including but not limited to life crises, technological and social change, immigration, economic and socio-political turmoil, and pandemics (Hogg, 2009, 2020). This group identification provides the individual with a sense of purpose, who they are, prototypical guidelines to follow, a value system, a simple dichotomy between “us” and “them”, and a basic understanding of world phenomena (Hogg, 2014, 2020; Hogg et al., 2013). Studies utilising this framework outline extremist collectives in different uncertainty-inducing scenarios (Hogg, 2020; Hohman et al., 2010; Mutallimzada & Steiner, 2023). For instance, Mutallimzada and Steiner (2023) analyse the mobilisation of Ukrainian fighters pre-2022 into the Volunteer Ukrainian Corps, showcasing how self-uncertainty is reduced through the paramilitary group’s high levels of prototypicality and entitativity in a conflict environment. In another study, Hogg (2020) outlines how the rising toxicity and existential threat narratives in far-right populist leadership create an effective environment for their supporter’s in-group affinity and anxiety reduction.

How does this relationship between group identity and reducing uncertainty relate to extremist mobilisation? Hogg argues that radical or violent movements are particularly effective at manipulating and recruiting insecure followers. These successful practices depend on the following principles, later used for the study’s deductive codebook (Hogg, 2014, 2020; Hogg et al., 2013):

1. *Group-centralism and entitativity*: A clear ideological foundation usually centred on ethnonationalism, xenophobia, or nativism. Entitativity—a group with a strong and united organisational structure—represents internal homogeneity and consensus, ritualised practices, a hierarchical structure, and closed membership boundaries (Hogg et al., 2010). Therefore, the group structures clarify who belongs and who does not, with a strict limitation encompassing group-centrality, forcing individuals to conform to the stipulated belief system and appropriate assemblages of communication.
2. *Confirmation bias and groupthink*: The formation of echo chambers of similar ideologies work together to discredit external information, ensure continuity and homogeneity within group discourse, and reinforce preconceived understandings (Hogg and Rinella, 2018). Individuals want to verify and validate their worldly experiences, which they pursue by creating a shared reality with other like-minded community members. Thus, we see a shift from individuality to collective social realities, combining different attributes, perceptions, values, and feelings into one coalescent being. The use and creation of conspiracy theories provide a simple example of this phenomenon in curating a set of familiar narratives and ideological foci.
3. *Prototypicality and the importance of leaders*: Leadership plays an essential role in the formation and maintenance of extremist groups. The more an individual identifies with the group’s leader (or prototypical members), the more imperative that collective is to their sense of self (Hohman et al., 2010). Moreover, followers pay particular attention to the prototype—the base characteristics—of the community’s leading members, basing their actions and words on them. Thus, we will often see a mimicking or rehashing of sayings of popular political leaders amongst their constituents (for example Donald Trump’s “drain the swamp”).
4. *Existential threat*: The self-construction of existential threats is used to purposefully challenge the group’s ideological certainties to create a common cause and produce violent mobilisation to protect their community. Importantly, the group believes that its sovereignty and livelihood are both supreme and simultaneously challenged by the actions of outsiders, which function to destroy their shared identity and way of life (Hogg, 2020). Therefore, radical measures—often in the form of hate-filled online and offline abuse—are necessary to reinforce the collective strength of the in-group (see Esses et al., 2013; Federico et al., 2013) while also justifying (digital) violence against the existential threat.

Therefore, the theory suggests how groups utilising authoritarianist, anti-democratic, exclusionary, or holistic nationalist principles can successfully recruit insecure individuals, especially throughout the vulnerability-inducing events of significant socio-political and economic upheaval. By establishing the four pillars of Hogg’s uncertainty-identity theory, the study can utilise the concept as a basis for case selection and a deductive coding framework.

## Methodology, Case Selection, and the UK

I employ a mixed-methods approach of netnography and QCA to produce a comprehensive narrative for the varying UK-centric themes and pandemic-inspired content on Gab. The former offers an ever-adapting methodology to the continuous changes in online communications and is best conceptualised as a reconstituted ethnography—providing both a method for data collection and primary source fieldnotes—for examining social media platforms and their users (Kozinets and Rosella Gambetti, 2020). The latter research method offers a guiding process for evaluating thematic patterns of words, concepts, and narratives in right-wing extremist media (Schipper & Spekkink, 2015). This dual-qualitative research’s selection of the deductive (QCA) and supplementary (netnography) perspectives provides the experimental framework necessary for an investigation into the vast content ecosystem of far-right groups and political figures of the UK (Morse, 2010). With the large amounts of qualitative data screen captured in different visual and textual forms, QCA is an effective partnering method to systematically outline the far-right’s content (Kozinets and Rosella Gambetti, 2020). Conversely, where QCA fails to capture the complex dynamics between the content’s meaning and its impact on user experience, netnographers can record the participant’s missing emotions. This practice places the researcher as the non-participatory observer, recording the latent sentiments behind their sharing, posting, and commenting (Kozinets, 2015). Thus, these two methods work together to depict the nature of toxic communications on the far-right site Gab. By combining netnography’s experiential emphasis and systematic data collection methods with QCA’s coding mechanisms, the study can comprehensively examine the UK-orientated far-right content on the platform.

Gab offers an interesting look into far-right virtual content as the “home to free speech online” (Gab, 2023). This self-prescribed motto contributes to the “safe harbour” environment for the UK’s far-right influencers and followers to interact openly with one another, where many feel welcome to share their opinions without fearing any censorship or banning (Munn, 2022). Consequently, the article explicitly chose Gab for its public nature—there is no need to sign up for an account, and all data collected in the study is by simply searching for the subgroup or user. While ethics for examining online extremist or radical communities continues to be a grey area (see Conway, 2021), Gab’s “decentralised and open” publishing policy helps alleviate the public versus private ethical debate (Gab, 2023; Rosenberg, 2010). Moreover, this access type differs from other far-right fringe websites (for example Parler, Truth Social, Telegram, MeWe), whose content is hidden behind creating an account. Another consideration is the article’s use of far-right influencers, wherein I piggyback off Berger’s (2018) argument on public figure labelling. The author contends that the selected far-right influencers (in this article, Tommy Robinson, Jayda Fransen, and Mark Collett) are self-defining popular or political figures for the movement, thus warranting scrutiny and open identification (Berger, 2018; Conway, 2021). All other data from non-verified accounts is anonymised.

Specific choices are made regarding the field, the number of posts, the content form, and the data-gathering period. Herein, the five most followed UK-specific and content-centric subgroups and influencers on Gab (see Table 1) detail the study’s case selection. To capture the narrational foci, I manually collected their daily top-five posts. I specifically sought the content which most resonated with the platform’s users, arguing that the more interaction—likes, shares, and comments—the content receives, the more that post appeals to user engagement and their sense of collective (see Hagemann & Abramova, 2023). Gab’s ranking system pre-sorts this content, prioritising the posts as “popular”. Importantly, this technique ensures a fair and equal representation of the cases studied and a systematic collection method (Kozinets, 2015). Moreover, the data was collected daily between May and June 2022. This choice offers a fascinating lull period where the number of COVID-19 cases was relatively low compared to January–April of the same year (WHO Coronavirus, 2023). Moreover, the extended period provides the time to integrate and understand the cultural, symbolic, and ideological nuances of the UK’s far-right virtual community (Kozinets, 2015). However, daily content amongst the selected cases is inconsistent. Therefore, a random sample of 185 posts (the minimum number collected for every subgroup) was selected from each case to ensure equal representation. With the data collection parameters and period, the study analyses a sample of 925 trending posts. The data is initially coded via the outlined deductive framework with inductive subcodes retroactively added through the iterative coding process and recorded fieldnotes.

**Table 1:** Outline of cases selected, the user’s affiliation, and a brief description

Name	Affiliations	The user’s importance for UK-centric content
Mark Collett	Neo-Nazi, white supremacist, alt-right influencer, political activist, conspiracy theorist	Collett is a key-conspirator in British far-right narratives and founded the White Nationalist group Patriotic Alternative. He remains closely tied to other white supremacists and targets many minority groups (Jews, Blacks, Muslims, and LGTBQ)
Tommy Robinson	Anti-Islamist activist, political activist, ultra-nationalist, conspiracist	Robinson is the co-founder of the English Defence League (EDL) and has one of the largest UK followings on Gab. A vocal figure in the media, known for inciting far-right violence
Britain First	Political party, British fascist, anti-multiculturalist, xenophobia	The most active UK far-right political party on Gab. They describe themselves as “a patriotic political party that fights to put our own people first!” Officially re-registered as a political party (2021)
#Brexit GB	Gab group, ultra-patriotism, anti-vaccine, anti-migration	The open group offers a public forum for users to discuss “the state of our nation and the future of our indigenous British people in the twenty-first century”. Provides a perspective from the general populace
Jayda Fransen	Political activist, anti-Islamist activist, self-described extremist	Fransen is the leader of the British Freedom Party, using religious justifications for her far-right narratives. A former member of Britain First, she broke off in 2021 to create her own political party

Finally, why should readers care about the far-right mobilisation of UK users? In the offline domain, mobilisation has turned violent during the pandemic, with scholars arguing that far-right extremism presents an ever-growing threat in the region (Jupp, 2022). For the link to the online world, however, the UK’s far-right manifestation on fringe social media, including Gab, is relatively muted and understudied compared to the literature for the USA, Germany, and Australia (see Dehghan & Nagappa, 2022; Peucker & Fisher, 2023; Schulze et al., 2022). Recent phenomena suggest the need for a more rounded look at “Anglospheric” online far-right content. As a backlash to policies and de-platforming on Twitter and Facebook, many predominant UK political leaders and movements who advocate far-right ideologies turned to Gab as a bastion of free speech (Nouri et al., 2021). With official verification and unmoderated capabilities, these actors use fringe outlets to proliferate their hate-filled messages and ideologies to audiences in the country and beyond without the need to censor their content. These include anti-Islamist groups, the English Defence League, For Britain Movement, and Britain First, famous far-right figureheads such as Katie Hopkins, Patriotic Alternative leader Mark Collett, and political activist Tommy Robinson. Moreover, selecting the subgroups on Gab must sufficiently cover the study’s outlined definition. Therefore, Table 1 outlines the examined case, where it fits within the movement and its importance in the UK far-right space. As discussed, these cases provide the most consistent daily content and hold the largest presence of followers and content catering to the UK population. Thus, their various narratives and posts should help capture the prevalent toxic discourses promoting far-right online mobilisation in the region.

## The Far-Right’s Use of Insecurity: Inductive and Deductive Findings

The findings are separated via the deductive processes of Hogg’s uncertainty-identity theory. Each subcategory represents an essential microcosm of far-right mobilisation on Gab and is reinforced by the inductive netnographic findings, which co-organise the sample of 925 posts into a more detailed set of narrational descriptions. The following sections flow based on occurrences from existential threat (664), confirmation bias (575), entitativity (343), and prototypicality (203), representing a complex array of communication within this toxic online community.

### Existential Threat

Every far-right narrative utilising existential threats in the study represents a form of toxic communication. Whether these messages target specific minority communities or relate conspiracy theories to a contemporary issue, the platform’s hate-filled content seeks to promote societal fragmentation and animosities. COVID-19 remains critical in UK far-right discourse (22%) even during periods of limited infection rates (WHO Coronavirus, 2023). It is not the physical harm caused by the disease which worries the community but the purposefully curated adverse effects of the vaccine. Herein, getting the protective shot is a “death sentence”, conspiratorially facilitated (vis-à-vis governmental control) to “destroy the working-class people of Britain”. This facilitation involves experts, including doctors, politicians, media figures, and world leaders, creating distrust in who or who not to believe. Other forms of toxicity are more latent, targeting different facets of society as opponents of far-right values. For example, the left (22%) represents the perversity of Cultural Marxist society. The community believes that the “woke” liberal values of globalisation and multiculturalism, “which has brought in the flood of migrants”, are causing the country’s demise. Here, it is easy to see how different harmful narratives can spread against out-groups. The left, whether the LGBTQ community, liberal politicians, minority groups, migrants, or Jews, are said to be actively destroying the UK’s culture, white children, ethnicity, and world status. Moreover, Islamophobic (20%) rhetoric often receives similar accusations. Labelled as “destroyers of western values”, Muslims take on a variety of different threats, from “rape-fugees” or the “rapists of Britain”, terrorist plotters or sympathisers, financially demanding “leeches”, and destroyers of British values. These interlinked toxic narratives form the majority (72%) of UK-centric Gab communications, showcasing the *need* for existential threats for the far-right community.

However, according to the UK-orientated collective, there is no unified or sole danger. Instead, the far-right’s toxic narratives target material and immaterial topics (see Table 2), resembling a hodgepodge style of content that oscillates depending on the hot issue or ontological insecurity at the time. For instance, when a Pfizer report on vaccine efficacy is released to the public, it is often followed by anti-vaccine sentiments and conspiracies about its harms. Another common phenomenon is the derogatory referrals to “the left” when linked out-groups (LGBT, feminists, pro-choice supporters) are protesting, depicting them as “evil” and “demented” members of society. Therefore, UK-centric far-right content is reactive and dependent on news sources for material. Subsequently, these labelled threats do not exist within a vacuum. Rather, they are frequently used in tandem, forming an elaborate *threat corpus* which endangers their far-right community. These combinations include the use of COVID-19 as the government’s way to control the population (12%), unwanted immigrants and, by extension, Muslim migrants who are actively participating in the Great Replacement (8%) or White Genocide of the native population, and the “backwards” nature of the left and Islam which threatens the well-being of “our children” (8%).

**Table 2:** Existential threat narratives in Gab’s UK-centric space

Existential threat	Frequency *f*	Frequency %
COVID-19 and vaccinations	148	22%
The left	144	22%
Islamophobia and migrants	130	20%
Government control	81	12%
The Great Replacement	52	8%
Defending the children	52	8%
Financial difficulties	31	5%
Mainstream media	15	2%
Anti-Semitism	11	2%
Total	664	100%

### Confirmation Bias

While existential threats illicit the talking points for most far-right narratives, confirmation bias offers the narrational background to perpetuate these messages. Subsequently, readers may ask how this Gab community represents and reinforces their ontological worldviews through the ecosystem’s toxic content milieu? The article argues, represented by Table 3, that the backbone of framing existential threats is the group’s “privileged” position in society to uncover hidden truths. This truth-seeking (44%) frame of mind works in multiple ways. First, the position allows for the continual *creation* of new threats. If the far-right relies upon various sources of anxiety as their main talking points, then the user’s reality uncovering position allows for the endless introduction of novel problems. These conspiracies take numerous forms, including the reinterpretation of sudden adult death syndrome as a “common vaccine side effect”, monkeypox as the “next biological siege on the people”, and the Russian-Ukrainian conflict as a symptom of the “Globalist system” (13%). Therefore, as headlines shift to cover breaking stories, this far-right online space spins these details to create novel anxieties. Moreover, it reinforces the community’s established narratives on existential threats. For this echo chamber’s reinforcement, fighting amongst refugees in different EU countries showcases multiculturalism as dangerous, Muslim migration into the UK exhibits “the fall of Western Society”, and incorporating LGBT-friendly material in schools is the left’s attempt to “indoctrinate and brainwash” children. These certainties are often strengthened by auditory and visual content, with blurry and cut-up videos created as a way to cement truth. Finally, facts are manipulated to emphasise the “anti-white system” the group currently faces. Part of the larger victimhood narrative (37%), their white privilege to truth paradoxically highlights their “failing” societal position. Thus, the online community frequently illustrates that the “indigenous” British population are “second-class citizens”, where being “proud to be white” or “born British” is a distinct disadvantage in society. This toxic discourse dichotomously establishes victimhood based on the out-group’s inherent and intended racism while forming the foundation for the in-group.

**Table 3:** How the UK’s far-right Gab community ontologically frames threats

Confirmation bias	Frequency *f*	Frequency %
“Truth” seekers	254	44%
Victimhood narratives	214	37%
The globalist system	73	13%
American influence and narratives	34	6%
Total	575	100%

### Entitativity

Gab’s UK influencers, groups, and users establish their community basis vis-à-vis two contrasting mechanisms: between who *we are* and who *we are not*. Both methods use distinct content forms but continue to rely on hate-filled narratives and conspiracies. Therefore, Table 4 can be rearranged into two inductively created categories: indigenous, patriots, protectors, we/us, Christianity, and the working class fall under who we are (68%), and anti-left, gender, and sexuality in who we are not (32%).

**Table 4:** In-group parameters of belonging in the British Gab space

Entitativity	Frequency *f*	Frequency %
Indigenous, patriots, and protectors	130	38%
Anti-left	64	19%
“We” and “us”	59	17%
Gender and sexuality	44	13%
Christianity	34	10%
Working class	12	3%
Total	343	100%

Building off the existential threats and confirmation biases dominating Gab’s content milieu, the UK’s far-right “indigenous” population see themselves as “this nation’s final hope” in protecting against “our country’s destruction”. However, styles of protection and patriotism (38%) range dependent on the case. Britain First politically campaigns with “battle buses”, travelling to minority-dominated areas to record themselves antagonising Muslims. These videos are later doctored to portray Islam’s “ill-fit” into Western Society and rally support as the victims of their violent responses. The other far-right influencers create insidious narratives about Muslims within this white supremacist framework. Robinson calls upon his followers to take to the streets to “fight back” against “Islamic terrorists” who are “raping white British girls” (and co-created a documentary titled *The Rape of Britain*). Collett follows a similar storyline, explaining in socioeconomic terms how “protecting our borders from ‘Asian’ migrants” will maintain the country’s cultural “Britishness”. Moreover, general evocations of “we”, “us” (17%), and Christianity (10%) offer simple identifiers for group belonging. Notably, religion is often exploited as a justification tool, where “Christian Nationalism” and “Muscular Christianity” provide activists with a “God-given right” to mobilise in the fight to “take back our country”.

Contrariwise, establishing the out-group or “who we are not” helps reinforce in-group identity. This labelling is a comparative process, where existential threat narratives on the “evils” or “backwardness” of “leftists” cement how not to behave. The same comparisons are prevalent in gendered and sexuality discourses. Herein, all gay men are equivalised to “sexual predators”, “paedophiles”, “child groomers”, and “weak”, dehumanising the LGBTQ community as lesser while stipulating the importance of manliness. Non-heterosexual communities also challenge the traditional nuclear family makeup and fail to “build the sole white communities” desired by the far-right. Therefore, Gab’s UK group centralism is built on conservative values of family, sexuality, male bravado, religion, and stability, which are bolstered by the threatening actions and characteristics of out-groups.

### Prototypicality

Although referral to prototypical behaviour in the study is comparatively limited (22%), the mechanism provides valuable insights into who UK users are willing to listen to (see Table 5). Perhaps unsurprisingly, the article’s selection of influencers employs Gab to self-promote (38%) content. A weekly activity is via live streaming, where the hosts provide a summary of events involving the latest existential threats. Herein, the audience reinforces in-group belonging through participation and can directly contribute to the conversation with any of their questions, suggestions, comments, or donations. Other forms of prototypicality involve the recirculation of experts, media personalities, and politicians (46%) advocating against the community’s insecurities. Thus, figures like Tucker Carlson, Jair Bolsonaro, and Viktor Orban, who are regularly in the media spotlight, are described as “anti-woke” or “based”, willing to voice their concerns about the vaccine, “illiberal” Western “lockdown policies”, sexuality and LGBT teachings in school, and the “brainwashing by mainstream media”. These influential far-right speakers provide a sense of legitimacy to the group’s concerns. Finally, evocating “the people” (15%) cast the community’s struggles as popular opinion. Thus, a common narrative is a worldwide unity and, by extension, a “non-minority” belonging, attempting to mainstream their cause. Threats are presented as a majority public concern, where everyday ordinary citizens are “waking up” to “fight for the people’s rights” and against “foreign invaders and rapists”.

**Table 5:** Leadership narratives and practices for Gab’s UK community

Prototypicality	Frequency *f*	Frequency %
Self-promotion	78	38%
Media and experts	65	32%
Ordinaries	31	15%
Politicians	29	14%
Total	203	100%

## The Patterns of Toxic Communication: a Discussion on Far-Right Mobilisation

Outlining the connection between populace uncertainty during the pandemic, toxic online communications, and far-right participation requires a deeper look into why toxicity is such an effective tool for mobilising support. While the results highlight the different forms of the UK’s content milieu of hate-filled narratives and ideologies, Hogg’s (2007, 2009) mechanisms, when observed individually, do not capture the complete picture. Instead, in its current form, the study depicts the isolated themes without demonstrating the efficacy of its proponents. While these findings are essential for updating the scholarly literature on the UK’s far-right hate-filled discourse, the community’s use of toxicity and insecurity is not new (see Agius et al., 2020; Munn, 2020; Pearson, 2019; Sakki & Castrén, 2022). Therefore, this section highlights a novel dynamic, showcasing the interconnections between the four analysed pillars and how they are employed in a cyclically toxic communication pattern to mobilise participants.

This relational use of uncertainty-identity theory’s mechanisms (see Table 6) portrays an intricate system of co-utilisation. Though other scholars have highlighted the importance of the individual components and their effects on different extremist groups (see Michael Hogg & Adelman, 2013; Hohman et al., 2010; Rast, 2015), I argue that it is in a combinational use which generates their effectiveness. Thus, predominant pairings between mechanisms help tell different stories of toxic communication styles on Gab. For instance, of the 575 posts connected to confirmation bias narratives, a massive 478 (83%) of these posts also mentioned one of the community’s existential threats (Slovic, 2020). Other connections are more limited but essential, including pairing prototypicality with an existential threat (51%) (Bai & Federico, 2021) and confirmation bias with entitativity (38%) (Yzerbyt et al., 2001). For example, posts focusing on Muslims often present two narratives, one of out-group threat and the other an affirmation of a white victimhood worldview (Doerr, 2021). Entitativity works with confirmation bias to cement in-group belonging (Hamilton et al., 2011), utilising discourse on truth to impose a self-righteous belief as protectors of the UK. Finally, prototypicality offers another tool to legitimise insecurity narratives, with predominant influencers advocating against the group’s existential threats (Hogg, 2020).

**Table 6:** Co-occurrence table showcasing the overlaps between Hogg’s uncertainty-identity mechanisms

	Entitativity	Existential threat	Confirmation bias	Prototypicality
Entitativity	X	191	131	55
Existential threat	191	X	478	103
Confirm. bias	131	478	X	79
Prototypicality	55	103	79	X
Total	343	664	575	203

**Fig. 1:**
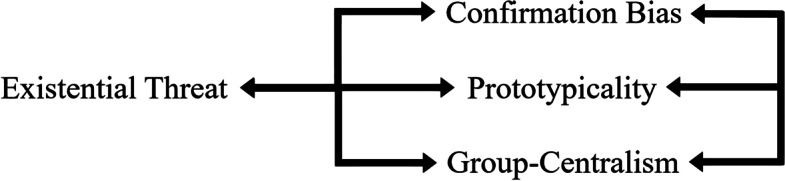
The co-utilisation relationship between Hogg’s uncertainty-identity mechanisms

Subsequently, these couplings all work in tandem, as illustrated by Fig. 1, with existential threat helping to establish and reinforce the group’s worldviews (confirmation bias), in-group belonging (entitativity), and prototypicality. However, the narrational process can both generate and structure societal insecurity. Herein, the community’s formulation of identity in these three latter mechanisms affects the threat corpus actively promoted on Gab. At the same time, confirmation bias, prototypicality, and group-centralism also interact with one another—on a more limited basis—to help reaffirm the user base’s collective self. Consequently, according to the findings, the UK-orientated community builds their mobilisation technique with these combinations, creating a cycle with two intermixing principles: the need for and creation of hate-filled content. Through demonstrating this connection, the work showcases the far-right’s need for different uncertainties to mobilise support, with these requirements necessitating the creation of toxic discourse directed against the outlined existential threats. The *Collective Anxiety* model helps explain this interdependent relationship and cycle.

The cyclically toxic communication model for rallying its user base, which this study calls *Collective Anxiety*, provides the illustrative framework connecting current societal uncertainties with online far-right mobilisation. While the previous scholarly use for the term ranges from research on “collective anxiety attacks” (Bartholomew & Victor, 2004), polarising topics and disputes in online spaces (Yang et al., 2021), and epidemic-induced mass hysteria (Bagus et al., 2021), the inspiration for applying the concept comes from Neal Curtis’ (2021) “Hate in Precarious Times: Mobilizing Anxiety from the Alt-Right to Brexit”. Curtis’ work demonstrates the relationship between the far-right’s intrinsic need for existential threat and societal anxiety with an accompanying feeling of anger and hatred, which connects individuals to a common cause (Curtis, 2021). I observe a similar phenomenon for communications on Gab, where the co-utilisation of Hogg’s insecurity-identity mechanisms behave in a cyclically reinforcing manner centred around far-right anxieties.

**Fig. 2:**
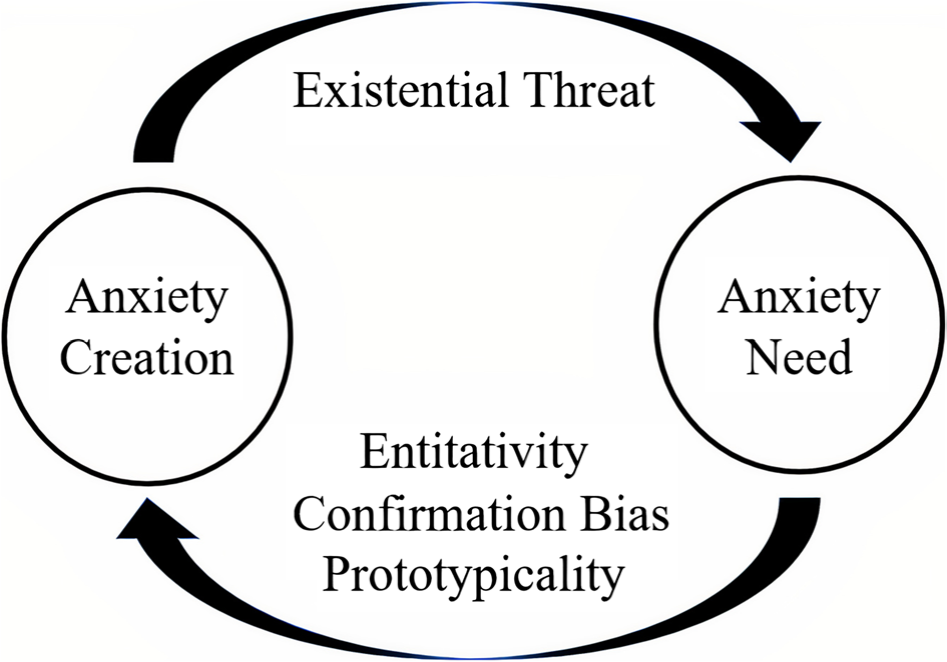
A cyclical model for mobilising support in Gab’s UK-centric online sphere

Building on this relationship, I illustrate a model (see Fig. 2) of online far-right mobilisation and the communities’ cyclical nature of harmful discourse. In order to create, promote, and maintain their community, the virtual collective establishes and manipulates common internal and external societal uncertainties (Agius et al., 2020). The community then exploits these anxieties and promotes membership and simple solutions (for example out-group scapegoats) to tackle the magnified problems (Rathje et al., 2021). Thus, the insecurities and solutions connect individuals to a common set of grievances and an ontological belief system for overcoming them (Castelli Gattinara et al., 2022). Moreover, these two dynamics build on one another, with mobilisation dependent on creating and expanding a threat corpus within their hate-filled content milieu (Marcks & Pawelz, 2020; Peucker and Fisher, 2023; Rone, 2022). Thus, *Collective Anxiety* is a cyclical and dichotomous relationship fuelling societal anxiety while providing answers and a sense of community to manage these uncertainties—an interdependent relationship between *anxiety-creation* and *anxiety-need*. Anxiety-creation refers to the far-right’s compilation of different existential threats, and anxiety-need the community’s reliance on Hogg’s three other pillars for in-group collectivity.

Readers may then ask what this cycle of toxic communication looks like in practice and if we can observe the differences between anxiety-need and creation? A few examples are discussed from other research in the field to illustrate how co-dependent discourse between these two modules is an effective tool for mobilisation. In Bliuc et al.’s (2019) work on collective identity changes, the scholars highlight a reactionary reformation of belonging due to an existential threat (Sydney Riots of 2015). Australia’s far-right exploits the intergroup conflict between whites and Muslims to change the community’s identifiers (creation) while making their “identity more inclusive to like-minded people” to mobilise and attract new followers (need) (Bliuc et al., 2019). In another example, researchers examine what the use of public anxiety means for garnering radical right political support. Their investigation outlines a dual process of far-right voter mobilisation between anger and fear, with societal insecurities initially prompting fear, eventually turning into anger towards out-groups (creation) while boosting support towards the in-group (need) (Vasilopoulos et al., 2019). Finally, the use of toxic masculinity to overcome ontological insecurity, as delineated by Agius et al. (2020), draws similar parallels to this study’s findings. As protectors of the “motherland”, the aggrieved loss of masculinity from economic, political, and societal insecurities breeds alienation and grievances amongst male far-right supporters (creation) (Agius et al., 2020). These gendered needs—protectors, patriots, and heteronormativity—subsequently create the collective and highlight the challengers to this sense of belonging (need). Thus, social and political movements for the far-right depend on different sets of uncertainties to prosper. At the same time, dependence is self-fulfilled by the labelling and promotion of existential threats and out-groups. This *Collective Anxiety* process is perpetually repeated, with new toxic narratives a necessity for further far-right mobilisation.

## What Next? Policy Implications and Conclusion

The article highlights the connections between societal insecurity, toxic online communications, and far-right mobilisation. The netnographic and QCA findings of 925 popular posts detail a complex set of narratives covering various topics. These include discussions on COVID-19, the vaccine, and implicating out-groups for their spread. Other hate-filled narratives revolve around the left, the LGBT community, migrants, and Muslims. Importantly, this content is framed with a particular goal: establishing an ontological worldview, an in-group belonging, or a prototypical leader to emulate. These deductive processes do not completely answer the research question, however. Instead, the article proposes a model—*Collective Anxiety*—of toxic cyclicity that better captures the effectiveness of their narratives in promoting mobilisation. With Gab’s UK-centric community dependent on both the need and creation of populace insecurities to attract and maintain its following, the far-right conjunctively utilises Hogg’s four pillars of identity formation in cyclical pairings. These combinations simultaneously exploit societal anxieties and promote membership in the group, offering simple solutions (scapegoating and health misinformation as examples) to the problem. Therefore, I argue that online far-right mobilisation depends on creating, propagating, and rehashing toxic communications within their community. Ultimately, this cycle of hate-filled narratives and belonging is proving especially harmful against minority groups—refugees, Muslims, the LGBT, and Jews—and to societal cohesion within the pandemic environment.

How can policy react to the identity-reinforcing pairings of toxic online communications, the cycle of *Collective Anxiety*, and the far-right’s targeted hate speech on Gab and other social media platforms? I contend that challenging these interconnected phenomena revolves around either directly breaking the toxic mobilisation cycle by removing online hate speech from these platforms or indirectly reshaping the community’s manipulation of these harmful narratives. For the first recommendation, different intervention strategies are currently implemented ad hoc (Blaya, 2019). These mechanisms include platform self-regulation through manual and automatic content removal, self-reporting for victims of online hate speech, and police or government-coordinated takedowns of content (Blaya, 2019; Williams et al., 2021). Herein, dealing directly with existential threat narratives—the vast majority of content observed in the study—would eliminate the paradigmatic connection for the co-utilisation of Hogg’s uncertainty-identity mechanisms. However, these strategies’ efficacy within the UK has been questioned, given the massive amounts of data to review (see Williams et al., 2021), and may present more challenges when dealing with fringe platforms like Gab. For instance, would Gab be willing to cooperate with police content takedowns, given its status as “the home of free speech online” (Gab, 2023)? The answer is likely no, or in an extremely limited capacity. In December 2022, Gab released a statement on the UK government’s attempt to remove a content creator for “racial hatred”, to which the platform’s CEO responded “get bent” (Gab News, 2022).

Suppose we cannot consistently challenge, block, or remove toxic narratives on Gab. In that case, we must provide a different solution to tackle the indirect or deep-seated mechanisms behind the far-right’s narrational manipulation of existential threats. Höffler et al. (2022) provide an extensive summary for understanding the precursors or vulnerabilities towards anxiety dependence, which overlap with many of the study’s findings. These factors can range from seeking group belonging for those who felt ostracised and rejected from mainstream society to fighting against perceived injustices or shared grievances and constructing a simple black-and-white worldview with out-group scapegoats (ibid.). Consequently, providing a catch-all solution for those experiencing *Collective Anxiety* mobilisation is not easy. However, prevention is also not impossible. The article concludes with a call for building *community resilience* against online toxicity. Instead of resolving complex societal uncertainties, we can create a multimodal online landscape with the appropriate resources to resist far-right virtual mobilisation (Gerrand, 2022). Within this call, emerging studies suggest that constructing resilience online must focus on stimulating an alternative mode of collectivity which promotes community bridging, cultural identity, and trust (Gerrand, 2022; McNeil-Willson, 2022). Uncertainty does not need to divide society; it can be a tool of collective unity, promoting wide-ranging, inclusive in-group values of mutual support (McNeil-Willson, 2022). The targeted approach is bottom-up, meaning local civil society participation could reconcile polarising narratives (anxiety-creation) and missing in-group mechanisms for those experiencing insecurities (anxiety-need). Not only would this challenge the far-right’s *Collective Anxiety* framework and existential threat reliance, but it would also counteract the community’s other dependencies on prototypicality, entitativity, and confirmation bias. Therefore, by implementing novel strategies for online resilience, we can create an environment where toxicity can no longer serve as the source of far-right mobilisation.

## Data Availability

The datasets generated during the current study are not publicly available due to the harmful nature of its content but are available from the corresponding author on reasonable request.

## Footnotes

1 This study defines *mobilisation* as the increased involvement and growth of the platform’s user base to promote a collective ideal, cause, or action.

2 This study defines *toxic communication* as any hate-filled online content directed against a particular group, race, organisation, or ideology that evokes negative and emotional reactions against the target populace.
